# Bridging logic and time: the need for conceptualization of ‘functional neurological disorder before readiness’

**DOI:** 10.1093/braincomms/fcag222

**Published:** 2026-06-11

**Authors:** Akira Hanazono, Keita Yasuda, Yui Sanpei

**Affiliations:** Department of Neurology, Akita University Graduate School of Medicine, Akita 010-8543, Japan; Department of Neurology, Akita University Graduate School of Medicine, Akita 010-8543, Japan; Department of Neurology, Akita University Graduate School of Medicine, Akita 010-8543, Japan

We read with great enthusiasm the editorial by Stone in *Brain Communications* introducing the functional neurological disorder (FND) special collection.^1^ We strongly resonate with the ‘gentle note of caution’ that as FND rightly enters the mainstream, we must not lose sight of clinical, physiological, and sociological dimensions in favour of an exclusively neurocentric approach. Building on this pivotal insight, we wish to elaborate on two structural challenges that clinicians face at the intersection of objective diagnostic logic and the patient’s subjective reality, and propose a conceptual framing to navigate them.

The first challenge involves mitigating the sociological risk of ‘narrative surrender’.^2^ The transition to diagnosing FND via objective positive signs (e.g. Hoover's sign, tremor entrainment) has fundamentally liberated patients from the stigma of a diagnosis of exclusion. However, as Stone has previously noted, not all functional symptoms—particularly functional sensory disturbances—possess reliable and specific positive signs.^3^ Furthermore, he has also noted the classificatory struggles surrounding comorbid sensory symptoms, such as pain, given the inherent limitations in applying objective positive signs to subjective suffering.^4^ A patient's experience autonomously alters over time depending on their relational environment. Sociologists warn that strictly re-appropriating this dynamic process into medical terminology causes ‘narrative surrender,’ potentially alienating their subjective suffering.^2^ The International Association for the Study of Pain (IASP) similarly underscores that a patient's reported experience must be inherently respected, independent of objective neurobiological correlates.^5^ As FND enters the mainstream, neurologists must consciously balance the empowering nature of positive signs with the epistemic humility required to validate unquantifiable subjective narratives.

The second challenge relates to the temporal asymmetry between the establishment of a diagnosis and the patient's ‘readiness for change.’ Once functional positive signs are identified, they serve as a time-independent, logical anchor for the neurologist's explanation. However, the patient's capacity to integrate this explanation progresses autonomously as a time-dependent process.^6^ Recent literature suggests that ‘readiness for change’ is one of the predictors of rehabilitation success.^7^ Furthermore, current pathophysiological models suggest that FND involves aberrant predictive processing, serving as an autonomic adaptation to overwhelming arousal.^8^ Stripping away these functional symptoms prematurely—before the patient has developed the interoceptive capacity—can lead to therapeutic failure.

To bridge the gaps between the patient’s subjective time and the objective logic of diagnosis, we propose a formal conceptualization of a clinical staging phase, termed ‘FND before readiness.’ In this phase, positive signs should be used only as reassuring evidence of structural integrity. During this period, we assume treatment might not be aimed at immediate functional change, but rather at cultivating a secure holding environment. Clinicians can utilize low-pressure recreational activities and non-directive, supportive dialogue to build therapeutic rapport,^7^ until the patient exhibits potential signals of readiness, such as curiosity about the symptom mechanism or the ability to repeat back the diagnosis in their own words.^9^ Importantly, creating this secure environment is not a mere delay; evidence from comorbid functional disorders, such as irritable bowel syndrome, demonstrates that an empathetic patient-practitioner relationship serves as a profoundly therapeutic intervention in its own right.^10^

In conclusion, FND care requires a time-embedded ‘farmer's philosophy’ rather than a control-oriented engineering approach. Our objective is to stabilize the soil and secure an environment that fosters the patient's autonomous change. Positive signs could be used not only to remove stigma but also to serve as a means of security while awaiting future readiness.

## Data Availability

Data sharing is not applicable to this article as no new data were created or analysed.
